# Epilepsy

**Published:** 1911-09

**Authors:** James N. Brawner

**Affiliations:** Atlanta, Ga.


### EPILEPSY.

By James N. Brawner, M. D., Atlanta, Ga.

Epilepsy is a disease that is always with us and will remain so, at least, until the science of eugenics has developed to a point where in civilized nations the unfit will not be allowed to procreate. Among barbarous people the weaklings usually die early, being unable to cope with the harsh environments, thus keeping the race strong and hardy. But as man becomes more civilized, his intellectual view broadens and his sympathy more acute, the diseased and degenerate members of society are preserved, housed, clothed and fed, and are allowed to roam, begetting their kind. The epileptic, in the great majority of instances, belongs to this class. In nearly all cases he is an individual who has inherited an unstable nervous system from one or both of his parents. There are exceptions, of course, but it is the rule.

Before taking up the mental condition of the epileptic, I wish to report some experiments I made on rabbits a few years ago. While inoculating rabbits subdurally with normal salt solution, I found that occasionally they would have a local spasm, which at times would develop into a general convulsion. This lead me to make the following experiments:

Experiment No. 1: Rabbits were trephined over the motor area for the right leg and were injeced subdurally with six to twenty drops of normal salt solution, the injections being made slowly. No symptoms occurred, neither motor nor in the general behavior of the animal. In other words, the injections were made so that there was but little increased pressure on the brain.

Experiment No. 2: Rabbits were trephined as in experiment No. 1 and injected rapidly with six to twenty drops of normal salt solution, causing a sudden increase in pressure on the motor area of the leg. In most cases there would be a spasm of the right leg, which would extend to the left and finally cause a general convulsion. In some instances where the pressure was slight the spasm would remain local and would not extend to the other centers. When plain water was used instead of the salt solution the same results occurred except that not so large a quantity of the solution was required. So here we have increased local pressure on the motor region, causing local spasm, and if the pressure is sufficient the irritation will cause the molecular explosions to extend to other centers, with a general convulsion and loss of consciousness.

Experiment No. 3: Rabbits were trephined over the anterior part of the frontal lobes. If the injections were made slowly no symptoms, either physical or motor, would occur, but if made rapidly there would be physical and emotional symptoms displayed. The rabbit would sometimes run as if being chased, or squeal as if in great distress. What is more interesting, if the injections were made very rapidly, the physical symptoms would be followed by a general convulsion and loss of consciousness. In many of these cases I am sure there was no direct pressure on the motor region, but was irritated from the molecular explosions in the silent area.

The above experiments show that increased local pressure rapidly produced on the brain will cause a local spasm, and if the irritation is great enough the disturbance will spread to other centers, even to the point of overthrowing consciousness.

Now these experiments give us some idea as to the origin of convulsive seizures that are due to trauma, gross brain lesions, etc., that have all the characteristics of true epilepsy, and in many instances cannot be clinically differentiated. The convulsions, frequently Jacksonian in type at first, are due to an irritation of the cortical centers, causing a molecular explosion of the neurons. We must remember that the site of the irritation may not be on the motor area, but on other portions of the cortex, the excessive nerve discharge passing along the higher associative tracts and finally involving the motor pyramidal cells. Such convulsions, due to some irritation, as a spicule of bone or a gumma, *will occur in a person who has inherited a normal molecular stability of the cortical neurons, and herein lies the difference between this type of epileptiform convulsions and true epilepsy.*

What we diagnose clinically as epilepsy, no doubt, depends on a variety of conditions, but the type of the disease which develops in the young showing no signs of gross brain disease is nearly always due to an inherited molecular instability of the neurons of some part of the cortex which manifests itself in periodical explosive discharges of nerve force. This defect is not necessarily in the motor cells themselves but may be in the silent regions, the motor cells becoming secondarily involved through the associative tracts. If the discharges are comparatively light and involving the psychical area, we have psychical epilepsy, the convulsion being absent. No doubt the blind, unreasoning fury so frequently manifested, is of this order, the discharge not being sufficient to cause a complete loss of consciousness, though the patient frequently remembers very little as to what occurred during such an attack.

The explosive discharge passing from neuron to neuron through the cortex knocks to pieces and destroys those associative tracts and connections that have developed at a late date in the evolutionary period of man's history, and herein lies the organic foundations of the mental manifestations of the epileptic. It is unnecessary to describe in detail the mental manifestations that occur in epilepsy, for the experienced physician has seen many cases, but I wish to pay special attention as to why he displays the symptoms that he does. In studying the stability of the functions of the nervous system we must bear in mind the fact that the most stable and least likely to be overthrown are those that were acquired at the earliest age of the evolutionary period of man. Ever since the time when he crawled on his belly in slimy waters in the form of a mud fish, he would fight to defend himself, and this is one of the oldest and best organized faculties that he possesses. On the other hand, one of his most recent acquisitions is the capacity for abstraction and the ability to make wide and remote generalizations. Hence these, what we call his highest faculties are poorly organized and easily disarranged. No doubt they are represented in the cortex by comparatively unstable nerve matter in the form of recently acquired associative tracts. These tracts, being the most unstable, will be the first to suffer from any condition that causes an unusual amount of molecular disintegration. Now what occurs in epilepsy? There are periodic explosive discharges of nerve force, affecting, perhaps, every cell and tract in the brain, hence the latest formed and poorest organized association tracts will be the first to suffer. In other words, those high intellectual and ethical faculties that have been, comparatively speaking, recently acquired, will be the first to deteriorate. We need never expect an epileptic who has had many convulsions to be a learned astronomer or a great mathematician, or following any vocation calling for wide generalizations in thought. His faculties of restraint and inhibition will be deficient, allowing the baser instincts to have full sway. He will become furious on the least provocation and will fight his companions like a brute, using his fist, a stick, or any weapon that may be at hand. He is impulsive, often morose, and is dangerous when his temper is aroused. He is religious like the savage. He may be furious and fighting at ten o'clock and singing Psalms in church at eleven.

The mental symptoms depend a great deal upon the mental constitution of the patient. Some patients who are naturally timid never develop the aggressive tendencies seen in other epileptics. In some there is a gradual lowering of the general intellectual faculties, finally ending in dementia. One of the most interesting phases of the disease is the fact that some patients, either before or after an attack, will go about apparently conscious and in their lucid periods remember nothing as to what occurred. This is very interesting from the medico-legal standpoint.

Some patients have epilepsy for years and show only slight mental deterioration, especially when the attacks are far apart. These patients, no doubt, have inherited a nervous system of fair stability, and the disease most likely is due to some local irritation of the cortex.

The treatment of epilepsy is very important. After the epileptic habit has been established, a permanent cure of the attacks need not be expected. The interval between convulsions may be lengthened by proper care and treatment and every effort should be made in this direction, for every attack brings the patient nearer to a state of dementia. In children, the cause of the attacks should be carefully sought. If by exclusion we find that the attacks are due to an inherited instability of the cortical cells, the probability of a permanent cure is slight. But if some extraneous irritation is found, as an intestinal intoxication or pressure on the brain that can be removed, then the possibility of a cure is very much greater. We must always study each individual patient and try to determine whether the attacks will occur under any conditions of life, and those cases where there is fair stability, and the convulsions may be lessened by removing extraneous sources of irritation, and those where the patient has a normal nervous system and the attacks are due to some local disease involving the cortex. Of course, where external irritations are found, they should be removed, if possible.

Where the habit has been formed the diet should be regulated, the amount of salt taken reduced, and the bromides administered. I have found no drug that will diminish the number of attacks as the bromides, though they must be given judiciously. It is very important to keep the digestive organs in as good condition as possible.

Epileptics should invariably be watched as to their mental condition. Many cases give very little trouble, but we never know when they may develop a psychosis that will be dangerous to others. The impulsive character of the attacks makes them a menace to the unsuspecting public. Whenever an epileptic once shows an attack where he is dangerous to others, he should be placed under control, especially where he will have some occupation, as in an epileptic colony. When in an institution, he can be controlled, and given an occupation that suits him. He should be handled gently, and yet positively. By making his life as smooth as possible, lessening the ups and downs of life, regulating his diet and excretions and by proper medical treatment, we get the best results in true epilepsy.

